# Optimization of Buffer Additives for Efficient Recovery of hGM-CSF from Inclusion Bodies Using Response Surface Methodology

**DOI:** 10.22037/ijpr.2020.1101169

**Published:** 2020

**Authors:** Mina Ahmadian, Ali Jahanian-Najafabadi, Vajihe Akbari

**Affiliations:** *Department of Pharmaceutical Biotechnology and Isfahan Pharmaceutical Research Center, Faculty of Pharmacy, Isfahan University of Medical Sciences, Isfahan, Iran.*

**Keywords:** hGM-CSF, Inclusion body, Response surface methodology, Solubilizing, Refolding

## Abstract

Overexpression of human granulocyte-macrophage colony-stimulating factor (hGM-CSF) by *Escherichia coli* leads to formation of insoluble and inactive proteins, inclusion bodies. The aim of this study was to improve recovery of biologically active hGM-CSF from inclusion bodies. The effect of types, concentrations and pHs of denaturing agents and addition of reducing agents on the yield of inclusion bodies solubilization was evaluated. Next, various conditions were evaluated for refolding hGM-CSF using a two-step design of experiment (DOE) including primary screening by factorial design, and then optimization by response surface design. It was found that hGM-CSF inclusion bodies can be efficiently solubilized with 4 M urea and 4 mM β-mercaptoethanol, pH = 9. A response surface quadratic model was employed to predict the optimum refolding conditions and the accuracy of this model was confirmed by high value of R^2^ (0.99) and F-value of 0.64. DOE results revealed that sorbitol (0.235 M), imidazole (97 mM), and SDS (0.09%) would be the optimum buffer additives for refolding of hGM-CSF. Following refolding studies, the obtained protein was subjected to circular dichroism which confirmed correct secondary structure of the refolded hGM-CSF. The refolded hGM-CSF exhibited reasonable biological activity compared with standard protein. The approach developed in this work can be important to improve the refolding of other proteins with similar structural features.

## Introduction

Human granulocyte-macrophage colony-stimulating factor (hGM-CSF) is one of the most important hematopoietic growth factors which regulate the immune system (1). There are two different forms of hGM-CSF available for clinical applications, glycosylated (produced in yeast expression system) and non-glycosylated (produced in bacterial expression system) forms (2). Fewer complications have been reported for glycosylated form of hGM-CSF compared to the non-glycosylated one. However, the* in-vitro* non-glycosylated form shows higher biological activities and is up to ten times more potent than the glycosylated protein (3). Furthermore, non-glycosylated hGM-CSF is produced in bacterial system which has some advantages over eukaryotic expression system. 

Protein production in bacteria is faster and easier compared to other expression systems (eukaryotic cells such as yeast cells) so development and scaling up of the process is more convenient. However, like many other heterologous proteins, overexpression of hGM-CSF in *E. coli *leads to formation of insoluble proteins or inclusion bodies (4, 5). Inclusion bodies are insoluble molecules and mostly lack biological activity while only functional and soluble proteins could be applied in biotechnology (6). 

Various approaches have been proposed to recover biologically active protein from inclusion bodies (7). Common strategies to obtain active proteins from inclusion bodies include four steps of separating inclusion bodies from other bacterial components, solubilizing inclusion bodies, refolding solubilized proteins, and finally purifying refolded proteins by various chromatographic techniques. In the refolding stage, solubilizing or denaturing agent should be gradually removed from the solution. Dilution of solubilized proteins in the refolding buffer and dialysis of solubilized proteins against the refolding buffer are the most common methods for refolding and recovery of active proteins (8). Successful recovery of active protein forminclusion bodies is dependent on methods which are used for solubilization and removal of the denaturant and co-solute or additives which are added to the refolding buffer. Commonly used additives for refolding of inclusion bodies are chelating agents, buffers, detergents, osmolytes, amino acids , chaotropic agents , salts, and polyols (9).

Many studies evaluated the effect of refolding methods on recovery of functional proteins from inclusion bodies (10). However, there are few reports on the optimization of refolding buffer additives by a statistically designed experiment. The (statistical) design of experiments (DOE) is a quick and suitable method for planning experiments to understand and optimize relationship between independent and dependent variables (11).

In the present study, we evaluated the effect of different denaturing agents on solubilization of hGM-CSF inclusion bodies. In addition, the effects of various additives and their combinations on refolding the inclusion bodies were assessed using DOE. Next, the refolded hGM-CSF protein was purified using chromatographic method and *in vitro* biological activity of the obtained protein was determined by cell-based assay. 

## Experimental

*Expression of hGM-CSF*

His-tagged hGM-CSF was expressed in *E. coli* BL21(DE3) using auto-induction method as described previously (12). Briefly, pre-inoculum culture was grown in Luria-Bertani (LB) broth supplemented with 1% glucose at 37 °C overnight. This culture was added to a bioreactor containing 1250 mL of ZYP-5052 medium at a ratio of 1:1000. The culture was fed with supplementary medium containing glycerol, lactose, (NH4)_2_SO_4_, and Na_2_HPO_4_, as well as KH_2_PO_4 _after 7 h and incubated at 37 °C for further 18 h. At the end of expression time, the culture was centrifuged at 7,500 *g* for 10 min and the pellet was stored at -70 °C for future analysis. 

*Extraction of inclusion bodies*

The pellet was resuspended in cold solution buffer (50 mM Tris-HCl, 25% sucrose, 10 mM dithiothreitol (DTT), 1 mM NaEDTA, pH = 8.0) and then sonicated three times at 70 % amplification strength for 30 s with 2 s pulse-on and pulse-off intervals on ice. Then, lysozyme (1 mg/mL), benzonase (10 U/mL), and MgCl_2_ (2 mM) were added and the sample was vortexed. Next, lysis buffer (50 mM Tris-HCl, 1% Triton X-100, 10 mM DTT, 100 mM NaCl, pH = 8.0) was added and after a short vortexing, the sample was incubated for 45 min at room temperature. NaEDTA (15 mM) and MgCl_2_ (4 mM) were added and the sample was incubated for 30 min. Following reduction of the sample viscosity, it was centrifuged at 11,000 *g* for 20 min at 4 ºC, and the pellet was resuspended in cold wash buffer containing triton (50 mM Tris-HCl, 0.5% Triton X-100, 1 mM DTT, 100 mM NaCl, 1 mM NaEDTA, pH = 8.0) and sonicated on ice. Then, the suspension was centrifuged at 11,000 *g* for 20 min at 4 ºC and the pellet was resuspended in cold wash buffer (without triton) and sonicated on ice. Finally, the suspension was centrifuged at 11,000 *g* for 20 min at 4 ºC, and the supernatant was discarded. Washing the pellet with wash buffer lacking triton was repeated once more (13).

*Solubilizing of inclusion bodies*

Different concentrations (2, 4, 6 and 8 M) of urea and guanidine hydrochloride (GdnHCI)) as denaturant agents (14), and also their combination (2 M urea + 2 M GdnHCI, 4 M urea + 4 M GdnHCI) were used to solubilize extracted inclusion bodies. The effect of different pHs (5, 7, 9 and 11) and addition of DTT, n-propanol, and β-mercaptoethanol to solubilizing agent were also evaluated. Same amount of the inclusion bodies (obtained from previous step) was dissolved in different solubilizing agents and remaining insoluble particles were removed by centrifugation at 7500 *g* for 10 min, and then the supernatant was stored for further analysis.

*Refolding of Inclusion bodies*

Refolding the inclusion bodies was carried out in a 96 well plate using rapid dilution method. Twenty µl aliquots of the solubilized inclusion bodies were added to 180 µL of each refolding buffers. The plate was incubated at 4 ºC for 24 h and the turbidity of samples before and after incubation were evaluated by determination of absorbance at 600 nm using a microplate reader (Bio-Tek®, USA). The samples were centrifuged at 7500 *g* for 5 min to separate soluble (refolded) and insoluble (misfolded) fraction for further analysis. 

Based on screening of forty two buffer additives (Supplementary file, Table 1), seven additives and one buffer (50 mM Tricine, pH = 7) which showed more refolding efficacy were chosen. Then, the additives were added at different concentrations to Tricine buffer. The combinations of seven buffer additives were evaluated in 30 runs designed by Minimum Run Resolution V factorial design (Supplementary file, Table 2). Finally, three additives which had the highest positive effect on refolding were selected to design 15 experimental runs using Box-Behnken design Table 1 After analysis of the results, the optimum refolding condition was predicted by Design Expert® (version 7.0.0, Stat-Ease, Inc., Minneapolis, USA) and this condition was used for large scale refolding of hGM-CSF.

*Purification of hGM-CSF*

The solubilized hGM-CS and refolded hGM-CSF were purified with Ni-NTA affinity chromatography (hybrid condition) and size exclusion chromatography, respectively, as described below.

*Size exclusion* *chromatography*

HiLoad 16/60 Superdex 200 preparative grade column was used for purification of hGM-CSF. One mL of refolded hGM-CSF under optimum refolding condition was injected into the column and eluted at flow rate of 0.8 mL/min using a solution of 150 mM NaCl, and 50 mM Na_3_PO_4_, pH = 7.2 as mobile phase, and the fractions were collected every 1 min. 

*2. Ni-NTA affinity chromatography under hybrid (both denaturing and native) condition*

The solubilized and unfolded protein was applied to a chromatographic column containing Ni-NTA agarose (Invitrogen, USA) and incubated at 25 °C for 1 h by gentle agitation. The column was washed twice with the denaturing binding buffer (8 M Urea, 20 mM NaH_2_PO_4_, 500 mM NaCl, pH=7.8), and twice with the denaturing wash buffer (8 M Urea, 20 mM NaH_2_PO_4_, 500 mM NaCl, pH=6.0). Finally, the resin was washed four times with the native wash buffer (50 mM NaH_2_PO_4_ and 0.5 M NaCl, 20 mM imidazole, pH = 8.0) and hGM-CSF was eluted from column using native elution buffer (50 mM NaH_2_PO_4_, 0.5 M NaCl and a gradient of imidazole concentration ranging from 100-400 mM, at pH = 8.0).

*Analytical methods*

Protein samples were analyzed by 15% sodium dodecyl sulfate polyacrylamide gel electrophoresis (SDS-PAGE) and the intensity of specific bands were determined using TotalLab TL120 software (Nonlinear Inc, Durham NC, USA) to estimate the amount of proteins (different concentrations of bovine serum albumin were used as standards) (15). The concentrations of refolded and purified hGM-CSF were also determined according to the Bradford method. In addition, the secondary structure of refolded hGM-CSF was evaluated by circular dichroism using a J-715 spectropolarimeter (JASCO, Japan).

*Bioassay*

The biological activity of the refolded hGM-CSF was determined based on hGM-CSF induced cell proliferation. In brief, human promyelocytic leukemia cells, HL-60, and human leukemic monocyte lymphoma cells, U937, cells were seeded in 96-well plate (16). The next day, various concentrations (1-100 pg/mL) of the refolded hGM-CSF and standard hGM-CSF (R&D systems, USA) were added to different wells and further incubated for 48 h. Then, to evaluate cell viability, 20 µL of MTT [3-(4,5-dimethyl-2-thiazolyl)-2,5-diphenyl-2H-tetrazolium bromide] solution was added to each well and kept at 37 °C for 3 h. Finally, the well contents were replaced with 150 µL DMSO to dissolve formazan crystals and the absorbance was read at 570 nm by microplate reader (Bio-Tek®, USA).

## Result and Discussion

*Solubilizing of inclusion bodies*

**Figure 1:**
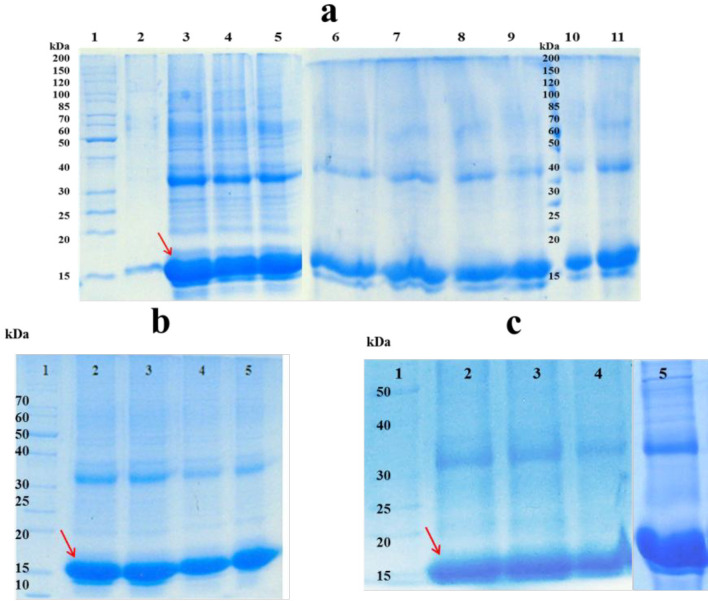
Soluble fractions after solublization of hGM-CSF inclusion bodies; a) effect of different concentrations of Urea and GdnHCl and their combinations on solubilizing inclusion bodies: Lane 1: protein marker (26614); Inclusion bodies were dissolved in 2, 4, 6 and 8 M Urea (Lanes 2-5). Inclusion bodies were dissolved in 2, 4, 6 and 8 M GdnHCl (Lanes 6-9). Inclusion bodies were dissolved in 2 M Urea + 2 M GdnHCl (Lane10), 4 M Urea + 4 M GdnHCI (Lane11).; b) Lane 1: protein marker (26614); Inclusion bodies were dissolved in 4 M Urea at pH = 11, 9, 7, 5 (lanes 2-5). c) Lane 1 protein marker (26614); Inclusion bodies were dissolved in urea 4 M at pH = 9 (lane 2); Inclusion bodies were dissolved in 4 M Urea at pH = 11 supplemented with different additives: 4 mM DTT (lane 3); 4 mM n-propanol (lane 4); 4 mM β-mercaptoethanol (lane 5).

To solubilize the inclusion bodies, different concentrations of urea, GdnHCI, and combinations of them were used. As shown in Figure 1.a, higher amount of hGM-CSF was dissolved when 4 M urea was used as solubilizing agent. Previous studies reported that efficiency of solubilizing with urea or GdnHCI is dependent on the solvent concentration and usually high concentrations of them (*i.e.* 6 M GdnHCl and 6-8 M urea) are required to completely solubilize inclusion bodies (17, 18). However, application of lower concentrations of urea (2-4 M) to solubilize inclusion bodies was also reported by some studies (19, 20). Similarly, in the present work, solubilizing of inclusion bodies was performed under mild denaturing condition which could preserve secondary structure of the protein, keep the protein in a partially folded state and facilitate the refolding step. Furthermore, high concentrations of the denaturing agent may lead to chemical modifications of protein (*e.g.,* carbamylation) (21).

Since solubilizing efficiency of urea is highly pH-dependent (22), different pH conditions for solubilization of hGM-CSF inclusion bodies were assessed and the optimum pH was found to be 9 (9 ≥ 11 ˃ 5˃ 7) (Figure 1.b). In the present work, the most efficient solubilizing was observed in 4 M urea at mild alkaline pH, although there are some reports for solubilizing of inclusion bodies using strong alkaline (≥ 12) or acidic (≤ 3) pHs with low concentrations or even no denaturing agents (23, 24). However, solubilization at extremes of pH may not be an appropriate strategy for the majority of proteins as many of them undergo irreversible modifications such as deamination and acid cleavage (25).

In addition to pH, the effect of reducing agents (DTT and β-mercaptoethanol) and organic solvents (n-propanol) were evaluated. As revealed by Figure 1.c, low concentration of β-mercaptoethanol significantly improved the solubilization of hGM-CSF inclusion bodies. This could be explained based on the fact that hGM-CSF molecule has two native disulfide bonds and nine cysteine residues, and this reducing agent can break non-native disulfide bonds and keep cysteine residues of the protein in their reduced state preventing protein misfolding and aggregation (26). Alternatively, other researchers reported solubilization of hGM-CSF inclusion bodies with 6 M GdnHCl supplemented with 10 mM DTT (27). However, DTT is more expensive and may interfere with protein purification process (*e.g.,* immobilized metal affinity chromatography) (28). 

*Refolding of inclusion bodies*

**Figure 2:**
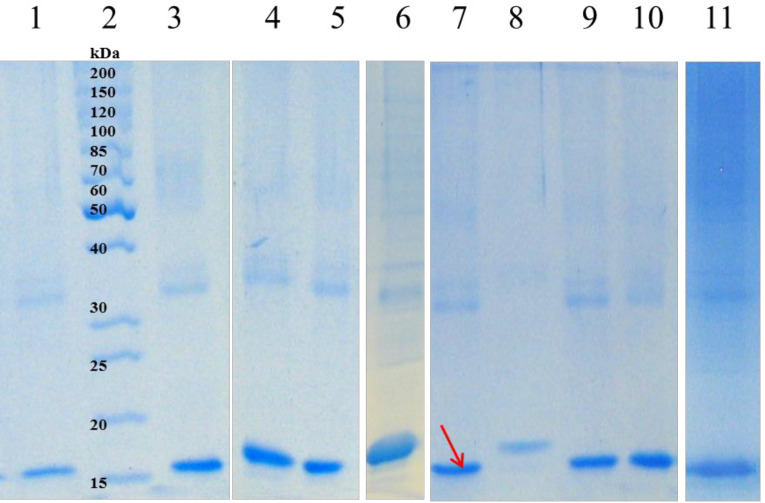
Soluble fractions after refolding of solubilized hGM-CSF inclusion bodies; Lane 2: protein marker (26614); solubilized inclusion bodies were diluted in refolding buffer containing: water (lane 1); 4 M Urea, 4mM β-mercaptoethanol, pH = 9 (lane 3); Citric acid (lane 4); Tricine (lane 5); Triton X-100 (lane 6); Imidazole (lane 7); Sorbitol (lane 8); SDS (lane 9); Urea (lane 10) or Ethanol (lane 11).

Among 42 buffer additives, sorbitol, imidazole, Triton X-100, SDS, urea, citric acid, ethanol, and Tricine were selected based on the change in turbidity of samples, densitometry of SDS-PAGE (Figure 2), and Bradford assay (having the highest protein concentration and the lowest turbidity change).

One of the most important parameters influencing the efficiency of refolding is pH of the refolding solution which determines the net charge of the protein. Proteins usually exhibit an improving solubility below and above of the isoelectric point (pI). Previous studies carried out the refolding of hGM-CSF (pI = 4.95) at a neutral to slightly alkaline pH (7.0-8.0) (27). Here, 50 mM Tricine, pH = 7 was chosen as the refolding standard buffer based on primary screening of 6 different buffers (Supplementary file,Table 1). Other seven additives were also evaluated in 30 combination experiments to determine the most important additives that influence the refolding. Three additives including sorbitol, imidazole, and SDS which had the most positive effect were chosen for further optimization Table 2. 

Fifteen experimental runs were designed by Box-Behnken to determine the optimum concentrations of these additives in refolding buffer. Table 3 shows the analysis of variance for response surface quadratic model describing the correlation between three additives and concentration of refolded hGM-CSF. This correlation could be represented as follows:

Concentration of refolded hGM-CSF (µg/mL) = 117.39 + 4.41 sorbitol + 24.94 SDS + 16.42 imidazole - 3.54 sorbitol×SDS + 25.96 sorbitol×imidazole + 9.33 SDS×imidazole - 36.79 sorbitol2 - 1.56 SDS2 + 12.25 imidazole2

**Figure 3:**
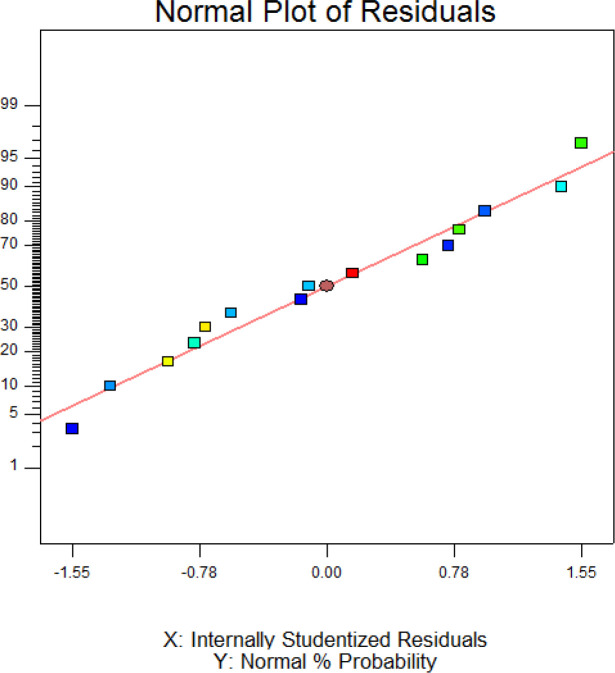
Normal (%) probability plot of the ‘studentized’ residuals for the model of hGM-CSF refolding

The Model F-value of 65.94 with a very low probability value (< 0.0001) Table 3 indicates that the model is significant. In adddition, SDS, imidazol, SDS×imidazol, sorbitol^2^, SDS^2^, and imidazole^2^ are signinficant terms (*p *< 0.05) in this model. The F-value of 0.64 and probability value of 0.6552 indicates the lack of fit is not significant in the model. As shown in Table 3, high value of “R-Squared” (0.9916) and “Pred R-Squared” (0.9247) are in resonable agreement with “Adj R-Squared” (0.9766) suggesting a good accuracy of the selected model. Figure 3 exhibited the normal probability plot of hGM-CSF refolding model. As it is shown, the data points follow a normal distribution which indicates this is a suitable and significant model for predicting hGM-CSF refolding at the different concentarions of these addititves. 

**Figure 4:**
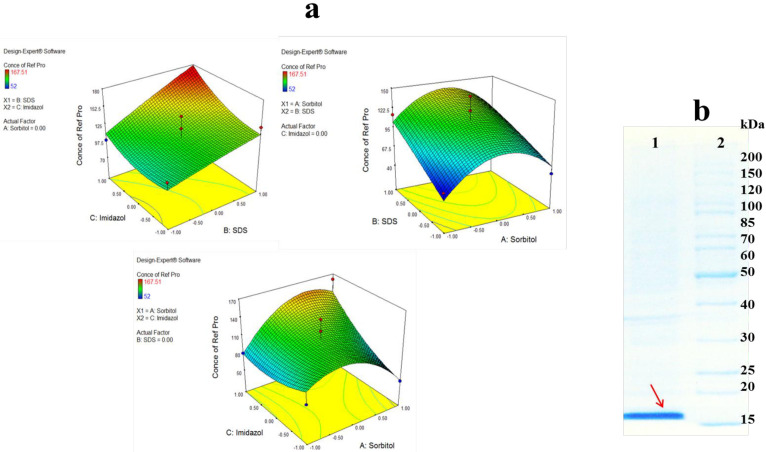
(A) Response surface plot for refolding of hGM-CSF shows the interaction between two factors in concentration of refolded hGM-CSF (µg/mL) by keeping other factor constant. (B) Large scale refolding of hGM-CSF with the optimum buffer; Solubilized hGM-CSF inclusion bodies were diluted in the optimum buffer additive condition (Lane 1) and protein marker (Lane 2)

Figure 4.a presents the two factor interactions between three buffer additives. As shown by the 3D surface graphs, refolding yeield of hGM-CSF increased significantly with an increase in SDS concentration, especially in the persence of high concentration of imidazole (*p *= 0.0054). Increasing concentrations of SDS at medium value of sorbitol led to an increase in hGM-CSF refolding, althougth the refolding yield reduced as the concentrriaon of sorbitol decreased or increased. SDS is mostly considered as a denaturing agent although in low concentrations (0.1-0.01%) it can decrease misfolding and formation of oligomers after elimination of denaturing agent (*e.g*., urea), and during refolding process (29). In the present study, SDS was used in the presence of sorbitol which is a polyalcohols co-solvent and osmolyte. Michaux *et al*., also reported some diol-type solvents can alter denaturing properties of SDS and combination of detergent and co-solvent may help refolding of proteins (30). Sorbitol can stabilize the protein conformation during refolding process. It was found that the effect of sorbitol on the stability of hGM-CSF was remarkably dependent on its concentration. Similarly, Xie *et al*., reported improvement of ribonuclease A stability by sorbitol in a concentration-dependent manner (31). They proposed general nonspecific bindings of co-solvent to refolded protein are responsible for stabilizing effect of sorbitol. Imidazole as an amino acid derivative can remarkably improve refolding of hGM-CSF. The refolding yield slightly enhanced when the concentration of imidazole increased at low concentration of sorbitol but this effect remarkably increased at high concentration of sorbitol. In consistent with our results, Shi *et al. *, reported that imidazole can significantly increase the refolding yield of green florescent protein (GFP). They suggested that imidazole acts like a chaperon molecule and refolding catalyst through enhancement of prolyl isomerization. However, they indicated that the additive is not a protein stabilizer and cannot prevent pH-dependent denaturing of GFP.

Based on DOE results, the optimum buffer for refolding of hGM-CSF was predicted as follow: sorbitol (0.235 M), imidazole (97 mM), and SDS (0.09 %). This optimized condition was used for large scale refolding of hGM-CSF (Figure 4.b) which yielded a concentration of 180 µg/mL hGM-CSF (corresponds to 18 mg refolded protein from 1 liter of bacterial culture). 

*Purification*

**Figure 5:**
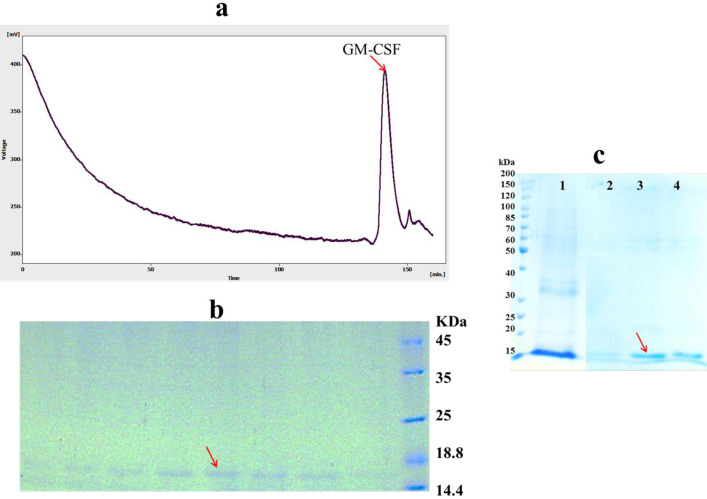
Purification of hGM-CSF (A) Gel filtration (Superdex 200) chromatogram of hGM-CSF. The arrow indicates the peak of eluted hGM-CSF. (B) The peak fractions of gel filtration (140-147 min) were analyzed by SDS-PAGE. The arrow indicates eluted hGM-CSF. (C) SDS-PAGE analysis of hGM-CSF purified using Ni-NTA column: Solubilized hGM-CSF before loading onto column (Lane 1); The fractions were folded and eluted under hybrid condition using the native elution buffer containing 100 (Lane 2), 200 (Lane 3) and 400 (Lane 4) mM imidazole

After protein refolding by dilution method, remaining host proteins and aggregated, misfolded and incorrectly folded intermediates of the target protein must be removed to increase the purity of refolded protein (32). To achieve this, refolded hGM-CSF was further purified by size exclusion chromatography. The refolded hGM-CSF was loaded onto the Superdex 200 column and eluted using a buffer solution of 150 mM NaCl, 50 mM Na_3_PO_4_, pH = 7.2. A clear peak was observed in the gel filtration profile (Figure 5.a) eluted in 140-147 min fractions. SDS-PAGE analysis was used to confirm the result of the gel filtration purification of hGM-CSF indicating the purity of eluted hGM-CSF (only one band was observed in 15 kDa region) in the isolated fractions (Figure 5 b). However, purification of refolded protein by Ni-NTA resin under native condition was not successful (data not shown) possibility due to lower accessibility of hexa histin tag of protein in refolded state (33). 

In addition to direct dilution method, solubilized proteins can be refolded and purified at the same time while bounded to a solid matrix like Ni resin. As shown in Figure 5.c, high amount of on-column refolded hGM-CSF was eluted in the fraction containing 200 mM imidazole. The purity of the refolded hGM-CSF was 90%. On-column refolding can prevent misfolding and aggregation of protein via decrease in undesirable intermolecular interactions between partially folded monomers (34). 

*Analytical and biological characterization of the refolded hGM-CSF*

**Figure 6:**
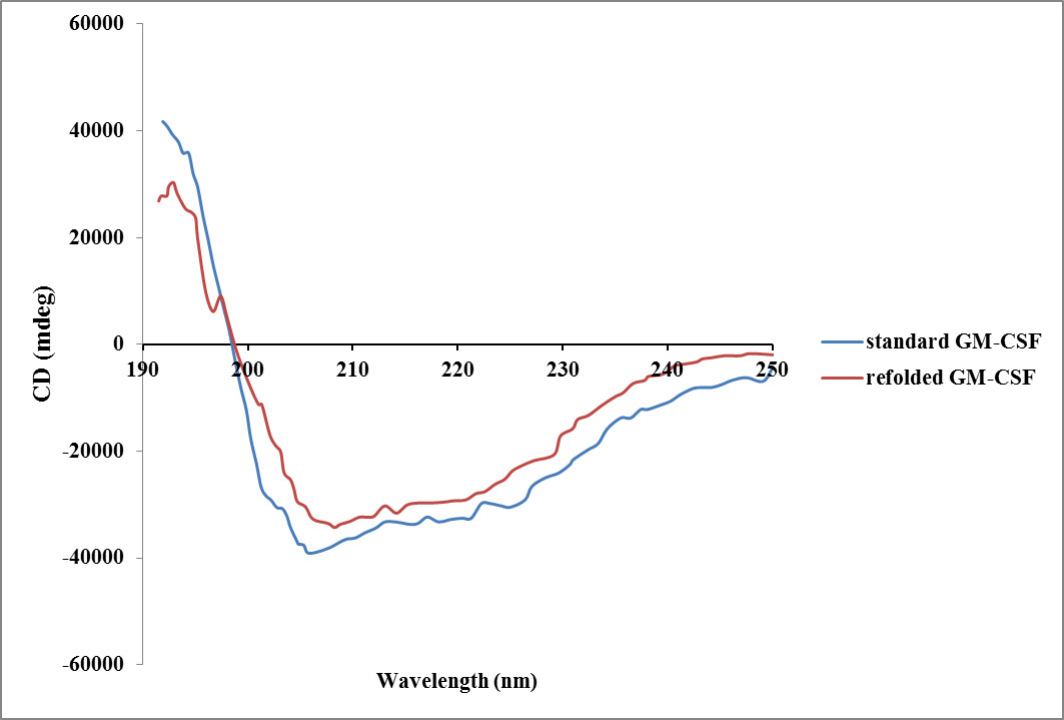
Circular dicroism spectra of refolded hGM-CSF compared with standard hGM-CSF

There are different structural- and functional-based methods to monitor refolding of protein. Some structural-based methods like circular dichroism (CD), fluorescence, and nuclear magnetic resonance (NMR) spectroscopies provide valuable information about the folding state (35, 36). Additionally, functional-based methods such as enzymatic activity measurement and biological assay can be applied to confirm correct recovery of tertiary structure of refolded protein (37). Crystallography analysis of GM-CSF revealed that it is a highly compact and globular protein consisting of a hydrophobic core. GM-CSF molecule contains four alpha helixes and two beta sheets (38). In the present study, the secondary structure of the refolded hGM-CSF using optimum buffer condition was evaluated by CD. The CD spectrum of refolded protein was very similar to that of the native protein (Figure 6) indicating correct formation of secondary structures (*e.g*, alpha helix and beta sheet) after refolding. *In-vitro* biological activity of the refolded hGM-CSF under the optimum condition was evaluated using HL-60 and U937 cell lines.

**Figure 7:**
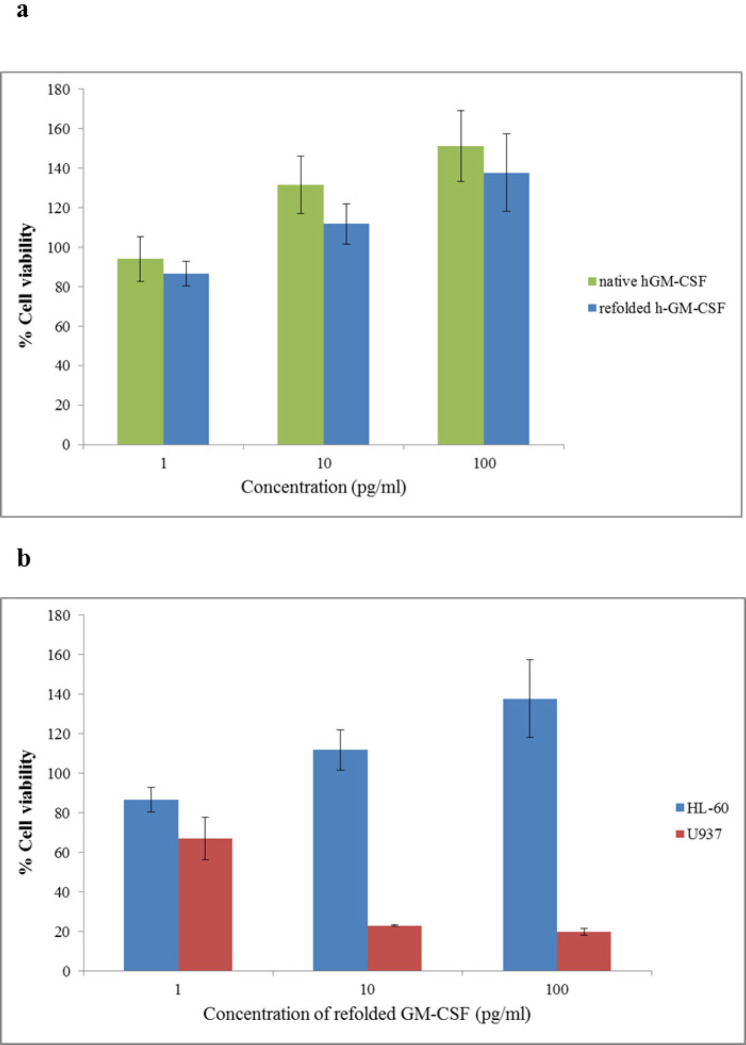
Biological assay of hGM-CSF using MTT assay (A) Effect of refolded hGM-CSF and standard hGM-CSF on viability of HL-60 cells (b) Effect of refolded hGM-CSF on viability of HL-60 and U937 cells. Cells were incubated for 48 h with different concentrations (1-100 pg./mL) of hGM-CSF. The vertical bars indicate the standard deviations (n = 4).

As it is shown by Figure 7, refolded hGM-CSF could stimulate proliferation of HL-60 cells in a dose-dependent manner. When HL-60 cells were incubated with 100 pg/mL of the refolded and standard hGM-CSF, cell proliferation increased 37% and 50%, respectively (the difference was not significant; *P *˃ 0.05). Refolded hGM-CSF exhibited similar biological activity to the native protein suggesting successful refolding of the protein. In this study, we evaluated the effect of hGM-CSF on the viability of two hematopoietic cell lines, HL-60 and U937. Interestingly, in contrast to its stimulatory effect on proliferation of HL-60 cells, hGM-CSF significantly inhibited the growth of U937 cells and this inhibitory effect was dose-dependent (*p *˂ 0.05). In agreement with our results, another research group reported inhibitory effect of hGM-CSF on the proliferation of U937 cells (39). They proposed that secretion of a soluble inhibitory molecule (probably tumor necrosis factor) by U937 cells following treatment with hGM-CSF is responsible for its anti-proliferative effects.

Different yields of hGM-CSF production in *E. coli* expression system have been previously reported. Earlier studies achieved a very low yield of protein expression (only 10% of total cell protein) due to toxicity of hGM-CSF to bacterial cell (40). However, there are some reports on successful application of fusion proteins (*e.g*., thioredoxin) for high yield expression of hGM-CSF (41). Fusion partner removal and further purification steps are needed for this strategy to prevent contamination of the product with a partially cleaved protein. Furthermore, a few additional amino acids are left by most of enzymatic cleavages interfering with bioactivity and immunogenicity of the protein (42). Therefore, fusion-tag approach is more time-consuming and expensive and it is not a common industrial bioprocessing method particularly at large scales. Alternatively, soluble and native protein can be obtained by refolding inclusion bodies. Thomson *et al.* reported successful refolding of hGM-CSF from inclusion bodies and they obtained a yield of 7 mg refolded protein per liter of bacterial culture (27). In the present study, we applied the systematic optimization of refolding additives which resulted in the highest yield of refolded and bioactive hGM-CSF from inclusion bodies (18 mg from 1 liter of culture) to date. The procedure developed in this work can be applicable to improve the refolding of other proteins with similar structural features. 

**Table 1:** Box-Behnken experimental design of 3 factors (refolding additive) at 3 levels (concentration).

**Concentration of refolded ** ** hGM-CSF** ** µ(g/mL)**	**Tricine** **(mM)**	**Imidazole or C (mM)**	**SDS or B (%)**	**Sorbitol or A (M)**	
146.5	50	50	0.05	0.25	Run 1
160.65	50	100	0.05	0.5	Run 2
127.42	50	50	0.05	0.25	Run 3
99.27	50	100	0	0.25	Run 4
107.33	50	0	0	0.25	Run 5
167.51	50	100	0.1	0.25	Run 6
53.66	50	0	0.05	0.5	Run 7
55.88	50	50	0	0	Run 8
80.12	50	100	0.05	0	Run 9
138.24	50	0	0.1	0.25	Run 10
52	50	50	0	0.5	Run 11
95.12	50	50	0.1	0.5	Run 12
113.17	50	50	0.1	0	Run 13
78.26	50	50	0.05	0.25	Run 14
76.97	50	0	0.05	0	Run 15

**Table 2:** Statistical analysis of buffer additives for hGM-CSF refolding by Minimum Run Resolution V factorial design. Buffer additives which had positive effect were highlighted

**Buffer additive**	**Stdized effect**	**Sum of squares**	**% of contribution**	**Significance**
Sorbitol	9.2	12.03	0.23	2
Imidazole	5.40	15.81	0.30	3
Triton	-4.48	106.83	2.00	6
SDS	4.90	492.43	9.21	5
Urea	2.79	296.14	5.54	7
Citric acid	-23.29	2011.35	37.63	1
Ethanol	-5.30	379.36	7.10	4

**Table 3:** Analysis of variances for response surface quadratic model developed by Box-Behnken design

**Source**	**Sum of squares**	**df**	**Mean square**	**F value**	**Prob > F**	
Model	0.073	9	8.146E-003	65.94	0.0001	Significant
A-Sorbitol	1.531E-004	1	1.531E-004	1.24	0.3162	Not significant
B-SDS	0.057	1	0.057	465.08	< 0.0001	Significant
C-Imidazole	7.381E-003	1	7.381E-003	59.74	0.0006	Significant
AB	9.000E-006	1	9.000E-006	0.073	0.7980	Not significant
AC	5.062E-004	1	5.062E-004	4.10	0.0988	Not significant
BC	2.704E-003	1	2.704E-003	21.89	0.0054	Significant
A^2^	2.285E-003	1	2.285E-003	18.49	0.0077	Significant
B^2^	2.061E-003	1	2.061E-003	16.68	0.0095	Significant
C^2^	1.533E-003	1	1.533E-003	12.41	0.0169	Significant
Residual	6.178E-004	5	1.236E-004			
Lack of	3.038E-004	3	1.013E-004	0.64	0.6552	Not significant
FitPure Error	3.140E-004	2	1.570E-004			
Cor Total	0.074	14				

R^2^ = 0.9916, Pred R^2^ = 0.9247 and Adj R^2^ = 0.9766.

## Conclusion

Here, we developed a simple and efficient method to recover biologically active hGM-CSF from inclusion bodies. The isolated inclusion bodies were successfully solubilized using low concentration of urea and a reducing agent at mild alkaline pH which could preserve native secondary structure of the protein and facilitate its refolding. Different buffer additives were screened and it was demonstrated that a combination of osmolytes, as a protein conformation stabilizer, amino acids, and their derivatives as refolding catalysts, and also, detergents as aggregation inhibitors can improve refolding the hGM-CSF. The optimum refolding condition was determined as sorbitol (0.235 M), imidazole (97 mM) and SDS (0.09 %) by response surface methodology. The refolded and purified protein exhibited correct secondary structure and full biological activity.
